# Exercise Modulation of the Myostatin–FOXO Pathway in Murine Models of Cancer Cachexia: A Systematic Review

**DOI:** 10.3390/medicina61112022

**Published:** 2025-11-12

**Authors:** Zahra Zare, Mahfoodha Al Kitani, Shahnaz Shahrbanian

**Affiliations:** 1 Department of Sport Sciences, Faculty of Humanities, Tarbiat Modares University, Tehran P.O. Box 14115-111, Iran; zahra.zare@modares.ac.ir; 2 Physical Education and Sport Sciences Department, College of Education, Sultan Qaboos University, Muscat 123, Oman; mkitani@squ.edu.om

**Keywords:** ubiquitin–proteasome, muscular atrophy, resistance training, colorectal neoplasms

## Abstract

*Background and Objectives*: Cancer cachexia is a debilitating metabolic syndrome highly prevalent in colorectal cancer (CRC), characterized by progressive skeletal muscle wasting. The myostatin–FOXO signaling pathway contributes to this process by activating the E3 ubiquitin ligases MuRF-1 and Atrogin-1. Exercise is a promising non-pharmacological strategy, but its effects on this pathway in CRC cachexia remain unclear. This review aimed to synthesize preclinical evidence on the impact of exercise on the myostatin–FOXO axis. *Materials and Methods*: A comprehensive search was performed in PubMed/MEDLINE, Scopus, Web of Science, and Science Direct from inception through August 2025. Eligible studies included murine CRC models (C26 or Apc^Min/+^) exposed to aerobic, resistance, or combined exercise interventions, with outcomes assessing myostatin, FOXO, MuRF-1, or Atrogin-1. Study quality was appraised using the CAMARADES 10-item checklist. *Results*: eleven studies met the criteria, with quality scores ranging from 6 to 8. Aerobic exercise, particularly voluntary wheel running, most consistently reduced MuRF-1 expression and systemic inflammation, whereas resistance and eccentric training exerted stronger inhibitory effects on FOXO and Atrogin-1. Myostatin was directly measured in two studies, yielding inconsistent results. Resistance and eccentric training promoted anabolic signaling (e.g., mTORC1), whereas aerobic protocols improved oxidative capacity. Variability in exercise type, intensity, and duration contributed to heterogeneity across findings. *Conclusions*: Exercise attenuates skeletal muscle catabolism in CRC-induced cachexia, mainly through modulation of the myostatin–FOXO pathway and downstream ligases. However, limited direct data on myostatin and methodological heterogeneity underscore the need for standardized protocols and translational studies. This review provides the first focused synthesis of exercise-mediated regulation of this pathway in CRC cachexia.

## 1. Introduction

Cancer cachexia is a multifactorial metabolic syndrome characterized by progressive skeletal muscle atrophy, with or without fat loss, driven by increased catabolism and impaired anabolic signaling, resulting in loss of muscle mass, strength, and physical function [1,2]. It affects up to 80% of patients with advanced malignancies, contributing to 20–30% of cancer-related deaths, with a notably high prevalence in gastrointestinal cancers like colorectal and pancreatic cancer [3,4,5]. Cachexia is more common and severe in older adults (typically over age 60) and males, who exhibit greater weight loss, muscle wasting, and worse outcomes compared to females [6,7]. Although aging, male sex, and inflammatory gene polymorphisms (e.g., TNF-α, IL-6, IL-10) increase susceptibility, the molecular drivers of muscle catabolism in CRC-induced cachexia remain incompletely understood [7].

Muscle wasting in cancer stems from tumor-derived catabolic factors and anticancer therapies (e.g., chemotherapy, radiotherapy), which exacerbate systemic inflammation, mitochondrial dysfunction, and proteolytic pathways in skeletal muscle [3,8]. These changes reduce treatment tolerance, lower quality of life, and worsen survival outcomes [9,10]. Given these multifactorial mechanisms, identifying the molecular regulators of muscle catabolism has become a research priority.

The myostatin signaling pathway plays a pivotal role in this process. Myostatin, a member of the transforming growth factor-beta (TGF-β) superfamily, binds to the activin type IIB receptor (ActRIIB) and activates mothers against decapentaplegic homolog 2/3 (SMAD2/3), thereby suppressing protein synthesis and enhancing catabolic signaling [11,12,13]. Elevated circulating myostatin in cachectic patients correlates with greater muscle loss and poorer prognosis [14].

Emerging evidence suggests exercise training may mitigate this catabolic pathway, offering a non-invasive strategy to preserve muscle mass [15]. Translational studies show that exercise, particularly resistance training, downregulates myostatin and FOXO activity, reducing MuRF-1 and Atrogin-1 expression, attenuating muscle catabolism, and preserving muscle mass [15,16]. Concurrently, exercise enhances anabolic signaling (e.g., mTORC1), promoting protein synthesis and metabolic homeostasis [17].

To our knowledge, no systematic review has specifically synthesized the effects of exercise on the myostatin–FOXO–Atrogin-1/MuRF-1 signaling axis in colorectal cancer (CRC)–induced cachexia. The focus on CRC is both clinically and experimentally justified. Clinically, CRC is one of the most prevalent malignancies worldwide, with cachexia affecting approximately 50–61% of patients, thereby contributing substantially to morbidity and mortality [18]. Experimentally, the C26 murine model of CRC is the most widely used and well-characterized preclinical model of cancer cachexia, offering reproducibility and strong similarity to human cachectic features [19,20]. Therefore, this systematic review aimed to synthesize preclinical evidence from murine models of colorectal cancer cachexia to determine how different exercise modalities (aerobic, resistance, and combined) modulate the myostatin–FOXO signaling axis and its downstream E3 ligases MuRF-1 and Atrogin-1.

## 2. Materials and Methods

This systematic review protocol was preregistered on the Open Science Framework (OSF) and is publicly available at https://doi.org/10.17605/OSF.IO/CDNFX (accessed on 18 September 2025). The review was conducted following the Preferred Reporting Items for Systematic Reviews and Meta-Analyses (PRISMA) guidelines [21].

### 2.1. Search Strategy

Four bibliographic databases were searched: PubMed/MEDLINE, Scopus, Web of Science Core Collection, and Science Direct from inception through August 2025. The complete Boolean search strings are presented in Supplementary Table S1 to ensure full transparency. An example of the Boolean logic used across databases was (exercise OR training OR physical activity) AND (myostatin OR GDF-8) AND (FOXO OR forkhead box O) AND (MuRF-1 OR Atrogin-1 OR MAFbx) AND (cancer cachexia OR tumor-induced wasting OR muscle atrophy).

### 2.2. Inclusion and Exclusion Criteria

Only original experimental studies conducted in animal models of colon cancer-induced cachexia were considered eligible for inclusion, provided they involved an exercise intervention such as aerobic, resistance, or combined training and reported molecular outcomes related to at least one of the following targets: myostatin, FOXO, MuRF-1, or Atrogin-1. Studies had to be published in English and available in full text. Articles were excluded if they used non-colon cancer models, lacked an exercise component, failed to report molecular markers following the intervention, or were non-original publications such as reviews, editorials, conference abstracts, or in vitro-only studies. Most included studies used murine models of colorectal cancer–induced cachexia, primarily the C26 or CT26 colon carcinoma implanted in BALB/c mice, while a few employed the Apc^Min/+^ transgenic model. These models consistently reproduce key cachexia features such as muscle wasting and body-weight loss.

### 2.3. Data Extraction and Analysis

Two independent reviewers (Z.Z. and S.S.) conducted the study selection and quality assessment. Discrepancies were resolved through discussion until full consensus was achieved. A third reviewer was deemed unnecessary due to full consensus between the two reviewers. Titles and abstracts were first assessed to exclude irrelevant records. Full-texts of potentially eligible articles were reviewed based on the inclusion criteria. Data on study design, cancer model, exercise protocol, and molecular outcomes (myostatin, FOXO, MuRF-1, Atrogin-1) were extracted.

### 2.4. Quality Assessment

The methodological quality of the included studies was evaluated using the 10-item checklist developed by the Collaborative Approach to Meta-Analysis and Review of Animal Data from Experimental Studies (CAMARADES) [22]. This tool assesses key domains, including peer-reviewed publication, temperature control, random allocation, allocation concealment, blinded outcome assessment, use of appropriate animal models, adaptation to the exercise apparatus, sample size calculation, ethical compliance, and conflict of interest statement. Each item was scored as 1 if present (“Yes”) and 0 if absent or unclear (“No” or “Unclear”), resulting in a maximum possible score of 10 per study.

### 2.5. Data Synthesis

Due to the substantial heterogeneity in experimental design, animal strain, tumor models, exercise modalities, and outcome measurements (e.g., mRNA, protein, or phosphorylated protein levels), a quantitative meta-analysis was not feasible. Accordingly, data were synthesized narratively using a structured approach consistent with the SWiM (Synthesis Without Meta-analysis) framework, emphasizing the direction and consistency of molecular effects rather than pooled effect sizes. This approach complies with PRISMA 2020 recommendations for heterogeneous preclinical data.

## 3. Results

### 3.1. Study Selection and Characteristics

**Figure 1:**
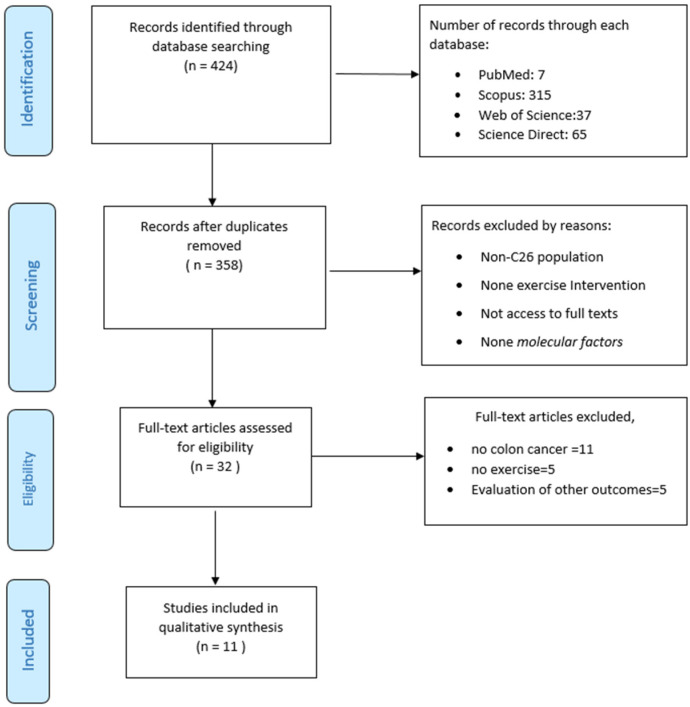
Diagram flow of outcomes of review.

The systematic search initially identified 424 records (PubMed = 7, Scopus = 315, Web of Science = 37 and Science Direct = 65). After removal of duplicates, 358 studies remained for screening. Following title and abstract screening, 32 articles were assessed for full-text eligibility. Of these, 21 articles were excluded (11 did not involve colon cancer models, 5 lacked exercise interventions, and 5 evaluated outcomes unrelated to molecular factors). Finally, 11 studies met the inclusion criteria and were included in the qualitative synthesis (Figure 1).

### 3.2. Characteristics of Exercise Programs

All included articles were original experimental studies conducted in murine models of colon cancer cachexia (C26 model). The exercise interventions varied considerably: some studies employed aerobic protocols (treadmill running or voluntary wheel running), while others implemented resistance training or combined modalities. Intervention durations ranged from acute to 8 weeks, with intensities adjusted according to animal capacity and study design. Molecular assessments were performed primarily in skeletal muscle tissue (gastrocnemius, soleus, quadriceps, or plantaris) using qPCR, Western blotting, to quantify myostatin pathway markers. A total of eleven preclinical studies were included, encompassing diverse murine models of colorectal cancer–induced cachexia, primarily the C26 carcinoma implanted in BALB/c mice, and the Apc^Min/+^ model. Exercise interventions varied in modality, duration, and intensity. Aerobic treadmill or voluntary wheel running protocols generally lasted 4–8 weeks at moderate intensity, whereas resistance training (ladder climbing) involved progressive overload up to 100% of body weight. Eccentric and combined protocols were also employed in some studies. The majority of investigations targeted the gastrocnemius or quadriceps muscles, assessing molecular regulators within the myostatin–FOXO–MuRF-1/Atrogin-1 axis. Aerobic exercise primarily reduced catabolic markers such as MuRF-1, while resistance and eccentric modalities elicited stronger suppression of FOXO and Atrogin-1 through anabolic signaling activation. These modality-specific adaptations highlight distinct but complementary molecular benefits across exercise types. The detailed study characteristics are summarized in Table 1 and Table 2.

### 3.3. Methodological Quality and Risk of Bias

The methodological quality of the included studies was assessed using the 10-item CAMARADES checklist [22]. As summarized in Table 3, quality scores ranged from 6 to 8, with a mean score of 7 out of 10. All studies were published in peer-reviewed journals and used appropriate animal models of colon cancer-induced cachexia. Blinding of outcome assessment was explicitly reported in one study, while the remaining studies did not specify this procedure. Randomization was generally described, but few studies detailed allocation concealment or random housing. Familiarization with exercise protocols and ethical compliance was adequately reported in most cases. Notably, only two studies included sample size calculations, indicating a general lack of statistical power considerations in preclinical exercise-oncology research [23,25]. Overall, methodological quality was moderate to high, though several domains of internal validity were underreported.

### 3.4. Outcomes

#### 3.4.1. Myostatin

Two studies directly assessed myostatin (GDF8), one via Western blot [23], and one via qPCR [31]. Tichy et al. (2025) observed elevated myostatin in tumor-bearing mice, which was significantly reduced after 4 weeks of VWR, paralleling attenuation of muscle wasting [23]. By contrast, Khamoui et al. (2016) reported no significant changes following aerobic or resistance training, despite improvements in IGF-1 signaling and muscle fiber cross-sectional area [31]. Given that only two studies assessed myostatin with conflicting results, no firm conclusions can be drawn. The regulation of myostatin by exercise remains unclear and likely depends on modality, duration, and methodological factors.

#### 3.4.2. FOXO

FOXO transcription factors were evaluated in three studies, with two using Western blot [25,27], and one using RT-PCR [29]. Dalle et al. (2024) and Fix et al. (2021) observed reduced phosphorylated or total FOXO levels after 16 days and 4 weeks of VWR, respectively, indicating suppression of catabolic signaling [25,27]. Tatebayashi et al. (2018) demonstrated that while C26 tumors increased FOXO1 expression, eccentric exercise (ECC-ES) did not significantly reverse this effect, although it promoted protein synthesis via mTOR activation [29]. Collectively, evidence shows that both aerobic and resistance modalities consistently suppress FOXO activity, contributing to muscle preservation.

#### 3.4.3. MuRF-1

All 11 studies assessed MuRF-1 expression (6 via RT-PCR, 4 via Western blot, 1 via RNA-seq). Sedentary tumor-bearing mice consistently exhibited elevated MuRF-1 levels. Exercise significantly reduced MuRF-1 expression in six studies, including those using voluntary wheel running [23,24,27,32], eccentric contractions [29], and high-intensity aerobic exercise [26]. Five studies reported no significant change: Dalle et al. (2024) with voluntary wheel running, attributed to short intervention duration (16 days) [25]; Hardee et al. (2018) after acute eccentric contractions, likely due to persistent inflammatory signaling (e.g., STAT3/NF-κB) [30]; Ranjbar et al. (2019) with combined exercise, despite improvements in muscle mass and strength [28]; Pin et al. (2015) with low-intensity endurance and Khamoui et al. (2016) with aerobic and resistance training no signinficant changes [31,33]. Overall, exercise frequently attenuated MuRF-1 expression, supporting its anti-catabolic effects in CRC cachexia models.

#### 3.4.4. Atrogin-1

10 studies assessed Atrogin-1 expression. Four studies [23,24,27,32] showed a significant reduction in Atrogin-1 following chronic voluntary or resistance exercise. In contrast, six studies [25,28,29,30,31,33] reported no significant changes in Atrogin-1 expression. Reductions were most consistently observed in studies with longer intervention periods or higher-intensity resistance protocols, whereas short-term or acute interventions tended to show no effect.

## 4. Discussion

**Figure 2:**
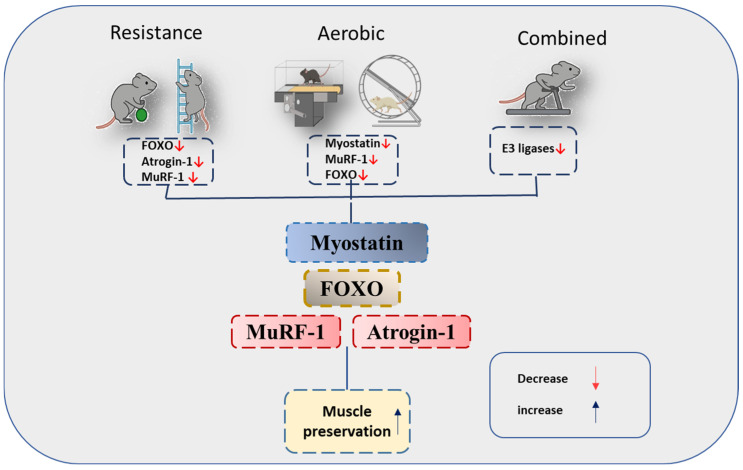
Exercise-induced inhibition of the myostatin–FOXO–MuRF-1/Atrogin-1 pathway in colorectal cancer cachexia.

This systematic review suggests that exercise interventions exert protective effects against CRC-induced cachexia in murine models, potentially involving modulation of FOXO signaling and its downstream effectors, MuRF-1 and Atrogin-1. The contribution of myostatin as an upstream regulator remains uncertain. A synthesis of the evidence suggests modality-specific responses. Aerobic exercise, particularly VWR, most consistently suppressed MuRF-1 expression and reduced systemic inflammation [23,24]. Resistance training and eccentric contractions had stronger effects on FOXO inhibition and more robust suppression of both MuRF-1 and Atrogin-1, likely mediated by IGF-1/AKT/mTOR activation [29]. High-intensity treadmill running not only reduced MuRF-1 but also showed anti-tumor effects [26], while combined exercise with pharmacological inhibitors demonstrated synergistic improvements in muscle preservation [32,33]. Collectively, aerobic exercise appears optimal for systemic and anti-inflammatory regulation, whereas resistance and eccentric modalities directly reinforce anabolic signaling and inhibit FOXO activity, and multimodal strategies may maximize benefits by targeting complementary pathways (A schematic is provided in Figure 2).

The role of myostatin as an upstream regulator remains less defined [34]. Only two studies directly examined this factor with divergent outcomes. Tichy et al. (2025) found elevated GDF8/11 levels in tumor-bearing mice, which were reduced by VWR, correlating with preserved muscle mass and cardiac function [23]. By contrast, Khamoui et al. (2016) observed no significant changes in myostatin with aerobic or resistance exercise despite improvements in muscle mass and IGF-1 signaling [31]. Given that myostatin activates SMAD2/3 and facilitates FOXO nuclear translocation, its suppression could represent a potential upstream mechanism contributing to the disruption of the catabolic loop that drives cachexia, though this remains speculative due to the limited and discordant evidence available [35,36]. The discrepancies across studies may reflect differences in intervention duration, intensity, or molecular assessments, suggesting the need for standardized and multiparametric analyses of myostatin in future research.

FOXO transcription factors emerged as a mechanistic hub linking myostatin signaling to the regulation of proteolytic genes. Exercise suppressed FOXO activity through AKT-mediated phosphorylation, preventing nuclear localization and activation of catabolic transcriptional programs [37,38,39]. Resistance training and eccentric contractions, which provide strong mechanical loading, were particularly effective in inhibiting FOXO3a [29]. Aerobic interventions such as short-term VWR also enhanced FOXO phosphorylation [25,27], although their effects appeared less consistent and more transient compared to resistance-based modalities. These observations underscore the importance of exercise intensity and loading characteristics as determinants of FOXO suppression.

Downstream, MuRF-1 and Atrogin-1 were both attenuated by exercise, though MuRF-1 was more consistently responsive. Significant reductions in MuRF-1 were reported across most interventions, including aerobic, resistance, and high-intensity protocols [23,24,26,27,29,32]. By contrast, suppression of Atrogin-1 was evident in fewer studies [23,24,27,32], indicating more variable responsiveness. The differential regulation of these E3 ligases may explain this pattern: MuRF-1 is influenced by both FOXO and NF-κB, which are strongly modulated by exercise, while Atrogin-1 is more dependent on AMPK/PGC-1α and may require prolonged or multimodal training for consistent suppression. Thus, MuRF-1 appears to be a more robust biomarker of exercise efficacy in cachexia [40,41].

Despite these consistent molecular findings, important limitations should be considered. All included studies were preclinical, using murine models such as C26, CT26, and Apc^Min/+^, which cannot fully replicate the complexity of human CRC-induced cachexia. The heterogeneity of exercise interventions, ranging from aerobic to resistance and eccentric training, varied in intensity, duration, and timing relative to tumor induction, making direct comparisons difficult and limiting conclusions on dose–response effects. Myostatin was rarely measured, preventing firm conclusions about its role as an upstream regulator. Importantly, because myostatin was directly measured in only two studies with inconsistent findings, any causal interpretation regarding its modulation by exercise should be made with caution. Variability in outcome measures, whether mRNA, protein, or phosphorylation status, may also have contributed to inconsistencies. Methodological shortcomings were common, including a lack of allocation concealment, an absence of blinded outcome assessment, and insufficient sample size calculations, raising concerns about bias. Furthermore, the predominance of male mice prevents evaluation of sex-specific responses, which may be clinically relevant given observed differences in cachexia severity between sexes.

Future studies should therefore aim to establish standardized exercise protocols with clearly defined parameters for type, intensity, duration, and timing to allow meaningful comparisons across models. Comprehensive evaluation of myostatin using advanced methods at multiple molecular levels is critical to clarify its role as a master regulator. Mechanistic experiments incorporating genetic or pharmacological modulation of myostatin, FOXO, or their downstream ligases in combination with exercise would provide causal insights. Integration of multi-omics approaches, including transcriptomics, proteomics, and metabolomics, could reveal additional pathways underlying the beneficial effects of exercise. Inclusion of both sexes in experimental designs is essential to determine sex-specific responses. Another limitation of this review is the possibility of publication bias. Since most available studies were small-scale preclinical experiments and often reported positive outcomes, negative or null results may have been underrepresented. A formal statistical assessment of publication bias (such as funnel plots or Egger’s test) was not feasible due to heterogeneity and the limited number of comparable outcomes. However, the potential influence of selective reporting cannot be excluded and should be considered when interpreting the overall findings. Finally, early-phase clinical trials testing patient-adapted protocols, such as moderate-intensity aerobic or low-load resistance training, are required to translate these preclinical insights into practice, with interventions tailored to cachexia severity, treatment side effects, and patient tolerance. A further limitation of the present review is that a quantitative meta-analysis could not be conducted due to the marked heterogeneity across animal strains, cachexia models, exercise modalities, and outcome measurements. Instead, we applied a structured narrative synthesis consistent with the SWiM (Synthesis Without Meta-analysis) framework, which emphasizes the direction and consistency of molecular effects rather than pooled estimates. This approach, while limiting statistical inference, enhances interpretive transparency and aligns with PRISMA 2020 guidance for preclinical evidence.

## 5. Conclusions

Exercise confers robust molecular benefits in CRC-induced cachexia, likely mediated through modulation of the myostatin–FOXO–MuRF-1/Atrogin-1 signaling axis. Aerobic exercise consistently reduces MuRF-1 expression and systemic inflammation, whereas resistance and eccentric training strongly suppress FOXO and Atrogin-1 through anabolic signaling, and multimodal approaches provide synergistic advantages. Given that myostatin was directly assessed in only two studies with discordant findings, this pathway should be interpreted as a plausible rather than definitive mechanism. Limited and inconsistent data on myostatin further highlight the need for additional mechanistic investigation. Overall, the evidence positions exercise as a promising, non-pharmacological strategy for managing CRC-associated cachexia, warranting translation into rigorously designed clinical trials.

## Figures and Tables

**Table 1:** Characteristics of the Retrieved Studies.

Author (Year)	Animal Model	Intervention	Comparison	Myostatin Pathway	Key Findings
Tichy et al. (2025) [23]	Male BALB/c mice, N = 10/group, 40 total (4 groups)	4 weeks VWR	Sedentary non- tumor-bearing, tumor-bearing	MuRF-1, Atrogin-1, GDF8 (Western blot)	Tumor-bearing: ↑ MuRF-1, Atrogin-1, GDF8/11, muscle/cardiac wasting. VWR: ↓ tumor burden, ↓ MuRF-1, Atrogin-1, GDF8/11, GDF15 VWR reduced myostatin signaling and preserved muscle mass.
Tsitkanou et al. (2025) [24]	BALB/c mice, N = 8–11/group, 72 total (4 groups)	4 weeks weighted VWR progressive loading (up to 4 g/day)	Healthy mice, tumor-bearing sedentary	MuRF-1, Atrogin-1 (RT-PCR)	C26: ↑ Atrogin-1/MuRF-1 VWR: ↓ Atrogin-1/MuRF-1, ↑ PGC1α Weighted running maintained fiber type and reduced catabolic genes.
Dalle et al. (2024) [25]	Male BALB/c mice, N = 5–6/group, 21 total (4 groups)	16 days of VWR	Healthy controls, sedentary C26 mice	MuRF-1, Atrogin-1, FOXO (Western blot)	VWR: ↓ p-FOXO, ↔ MuRF-1/Atrogin-1, Short-term VWR attenuated FOXO activation without significant changes in E3 ligases, suggesting an anti-catabolic effect via FOXO pathway
Jee et al. (2022) [26]	Male CDF1 mice, N = 10/group, 40 total (4 groups)	18 days high-intensity aerobic (90% max heart rate)	Healthy mice, tumor-bearing sedentary	MuRF-1 (RNA-seq)	Exercise: ↓ tumor growth, ↓ MuRF-1, ↓ CT26 proliferation (~20%) Short-term high-intensity aerobic exercise suppressed MuRF-1 expression and tumor growth, enhancing anabolic balance and overall survival in CRC-induced cachexia.
Fix et al. (2021) [27]	Male C57BL/6, Apc^Min/+^ mice, N = 6–10/group, 54 total (2 groups)	4 weeks VWR, 12 h fast	Sedentary MIN, B6 mice	FOXO, MuRF-1, Atrogin-1 (Western blot)	VWR: ↓ AMPK, MuRF-1, autophagy, Atrogin-1, FOXO, ↑ mitochondrial quality VWR suppressed proteolytic genes and improved mitochondrial quality.
Ranjbar et al. (2019) [28]	Male BALB/c mice N = 6/group, 24 total (4 groups)	6 weeks total (4 weeks pre- tumor + 11 days post-implantation); resistance: ladder climbing (20–50% BW); aerobic: motorized wheel (25 min/day, 4 days/week)	Healthy controls, tumor-bearing sedentary	MuRF-1, Atrogin-1 (RT-PCR)	C26 tumor: ↑ Atrogin-1, MuRF-1, ↓ muscle mass/strength. Combined exercise: ↔ Atrogin-1/MuRF-1 Combined (resistance + aerobic) training partially prevented muscle wasting and strength loss in C26- bearing mice by modulating autophagy and preserving mitochondrial function
Tatebayashi et al. (2018) [29]	Male CD2F1 mice, N = 5–7/group, 23 total (4 groups)	Eccentric contractions (acute, 14 sessions)	Tumor-bearing sedentary, healthy controls	Atrogin-1, MuRF-1, FOXO1 (RT-PCR)	↑ mTORC1 signaling (p-p70S6K, p-rpS6), ↓ MuRF-1, ↔ Atrogin-1/FOXO1/REDD1, preserved gastrocnemius mass ECC-ES enhanced protein synthesis through mTORC1 activation and partially reduced FOXO1-driven proteolysis.
Hardee et al. (2018) [30]	C57BL/6, Apc^Min/+^ male mice, N = 42 total	Single session eccentric contractions	Healthy controls	MuRF-1, Atrogin-1 (Western blot)	MuRF-1 ↔, Atrogin-1 ↔ Acute ECC activated AKT/ERK but not E3 ligases.
Khamoui et al. (2016) [31]	BALB/c mice, N = 16–17/group, 49 total (3 groups)	11 weeks (8 pre + 3 post tumor); treadmill 5–7 m/min; 5 days/week ladder 50–100% BW 3 days/week	Control, C26	Atrogin-1, MuRF-1, myostatin (RT-PCR)	RT + C26 and AT + C26: ↔ Atrogin-1/MuRF-1/ myostatin, ↑ IGF-1Ea (RT), ↑ p-mTOR (AT), ↑ CSA/strength/muscle mass Aerobic enhanced mTOR activation; resistance promoted regeneration with limited anti-cachectic effect.
Pigna et al. (2016) [32]	Male BALB/c mice N = 7–8/group	5 or 19 days VWR + AICAR or rapamycin	Tumor-bearing sedentary, Control	Atrogin-1, MuRF-1 (RT-PCR)	VWR: ↓ Atrogin-1, MuRF-1, counteracted muscle atrophy Short-term VWR suppressed proteolytic E3 ligases and mitigated tumor-induced muscle loss, independent of AMPK/mTOR modulation.
Pin et al. (2015) [33]	BALB/c, C57BL/6, male/female N = 6/group, ~24 total	2 weeks low-intensity endurance (acute) or 8 weeks (6 pre-tumor, 2 post-tumor) + EPO	Control, EX-only, EPO-only, tumor-bearing	Atrogin-1, MuRF-1 (qPCR)	C26: ↑ Atrogin-1, ↑ MuRF-1 in sedentary TB; EX-only ↔ Atrogin-1/MuRF-1 (no protection); EX + EPO ↓ atrophy, ↑ PGC-1α, restored oxidative fibers Low-intensity EX alone failed or worsened atrophy, but EX + EPO activated PGC-1α and improved muscle oxidative phenotype, partially suppressing catabolic genes.

AICAR: 5-Aminoimidazole-4-Carboxamide Ribonucleotide; AMPK: AMP-Activated Protein Kinase; Apc^Min/+^: Adenomatous Polyposis Coli Multiple Intestinal Neoplasia; AT: Aerobic Training; Atrogin-1: Atrophy-Related Gene-1 (also known as MAFbx); C26: Colon-26 Carcinoma Model; CSA: Cross-Sectional Area; CT26: CT26 Colon Carcinoma; ECC: Eccentric Contractions; ECC-ES: Eccentric Contractions with Electrical Stimulation; EPO: Erythropoietin; FOXO: Forkhead Box O Transcription Factors; FOXO1: Forkhead Box O1; FOXO3a: Forkhead Box O3a; GDF8: Growth Differentiation Factor 8 (Myostatin); GDF15: Growth Differentiation Factor 15; IGF-1Ea: Insulin-like Growth Factor 1, Extrahepatic Isoform; mTORC1: Mammalian Target of Rapamycin Complex 1; MuRF-1: Muscle RING-Finger Protein-1; p-FOXO: Phosphorylated FOXO; p-mTOR: Phosphorylated mTOR; p70S6K: 70-kDa Ribosomal Protein S6 Kinase; PGC1α: Peroxisome Proliferator-Activated Receptor Gamma Coactivator 1-alpha; PoWeR: Progressive Weighted Running; qPCR: Quantitative Polymerase Chain Reaction; REDD1: Regulated in Development and DNA Damage Responses 1; RNA-seq: RNA Sequencing; rpS6: Ribosomal Protein S6; RT: Resistance Training; RT-PCR: Reverse Transcription Polymerase Chain Reaction; VWR: Voluntary Wheel Running. ↑: increase, ↓: decrease, ↔: indicates no change or a stable level.

**Table 2:** Overview of Preclinical Studies.

Molecular Target	Exercise Type(s)	Number of Studies	Direction of Change	Summary of Evidence
Myostatin (GDF-8)	Aerobic, Combined	2	↓ or ↔	two studies assessed myostatin; aerobic and combined exercise tended to lower or maintain basal levels.
FOXO1/3	Aerobic, Resistance	3	↓	Most exercise modalities suppressed FOXO activation, reducing downstream proteolytic signaling.
MuRF-1 (Trim63)	Aerobic, Resistance, Combined	11	↓	Consistently downregulated across protocols; indicates inhibition of ubiquitin–proteasome activity.
Atrogin-1 (Fbxo32)	Aerobic, Resistance, Combined	10	↓	Closely follows FOXO suppression pattern; reduced expression observed in most models.

GDF-8: growth differentiation factor 8 (myostatin); FOXO—forkhead box O transcription factor; MuRF-1: muscle RING-finger protein-1 (Trim63); Atrogin-1: F-box only protein 32 (Fbxo32); ↓: decreased or downregulated; ↔: no significant change.

**Table 3:** Methodological Quality Assessment using the 10-item checklist of CAMARADES.

Author (Year)	1	2	3	4	5	6	7	8	9	10	Quality Score
Tichy et al. (2025) [23]	Y	Y	Y	N	N	Y	Y	Y	Y	Y	8
Tsitkanou et al. (2025) [24]	Y	Y	Y	N	N	Y	Y	N	Y	Y	7
Dalle et al.(2024) [25]	Y	Y	Y	N	N	Y	Y	Y	Y	Y	8
Jee et al.(2022) [26]	Y	Y	Y	N	N	Y	Y	N	Y	Y	7
Fix et al. (2021) [27]	Y	Y	Y	N	N	Y	Y	N	N	Y	6
Ranjbar et al. (2019) [28]	Y	N	Y	N	N	Y	Y	N	Y	Y	6
Tatebayashi et al. (2018) [29]	Y	Y	Y	N	N	Y	N	N	Y	Y	6
Hardee et al.(2018) [30]	Y	Y	Y	N	Y	Y	Y	N	Y	Y	8
Khamoui et al. (2016) [31]	Y	Y	Y	N	N	Y	Y	N	Y	Y	7
Pigna et al. (2016) [32]	Y	Y	Y	N	N	Y	Y	N	Y	Y	7
Pin et al.(2015) [33]	Y	Y	Y	N	N	Y	Y	N	Y	Y	7

(1) publication in a peer-reviewed journal; (2) statement of control of temperature; (3) randomization to treatment or control; (4) allocation concealment–blinded induction of cancer disease (i.e., concealment of treatment group allocation at the time of induction of ad); (5) blinded assessment of outcome; (6) appropriate animal species and ad models; (7) adaptation/familiarization to exercise apparatus; (8) sample size calculation; (9) statement of compliance with ethical regulations; and (10) statement regarding possible conflicts of interest. (Note: Abbreviations; Y = Yes, N = No).

## Data Availability

The data presented in this study are available within this manuscript.

## Supplementary Materials

The following supporting information can be downloaded at: https://www.mdpi.com/article/10.3390/medicina61112022/s1, Table S1: Comprehensive Search Strategy.

## References

[1] Tijerina A.J. (2004). The Biochemical Basis of Metabolism in Cancer Cachexia. Dimens. Crit. Care Nurs..

[2] Setiawan T., Sari I.N., Wijaya Y.T., Julianto N.M., Muhammad J.A., Lee H., Chae J.H., Kwon H.Y. (2023). Cancer cachexia: Molecular mechanisms and treatment strategies. J. Hematol. Oncol..

[3] Baracos V.E., Martin L., Korc M., Guttridge D.C., Fearon K.C. (2018). Cancer-associated cachexia. Nat. Rev. Dis. Primers.

[4] Fearon K., Strasser F., Anker S.D., Bosaeus I., Bruera E., Fainsinger R.L., Jatoi A., Loprinzi C., MacDonald N., Mantovani G. (2011). Definition and classification of cancer cachexia: An international consensus. Lancet Oncol..

[5] Ni J., Zhang L. (2020). Cancer Cachexia: Definition, Staging, and Emerging Treatments. Cancer Manag. Res..

[6] Zhong X., Zimmers T.A. (2020). Sex Differences in Cancer Cachexia. Curr. Osteoporos. Rep..

[7] Tan B.H., Fearon K.C. (2010). Cytokine gene polymorphisms and susceptibility to cachexia. Curr. Opin. Support. Palliat. Care.

[8] Coletti D. (2018). Chemotherapy-induced muscle wasting: An update. Eur. J. Transl. Myol..

[9] Blauwhoff-Buskermolen S., Versteeg K.S., de van der Schueren M.A., den Braver N.R., Berkhof J., Langius J.A., Verheul H.M. (2016). Loss of muscle mass during chemotherapy is predictive for poor survival of patients with metastatic colorectal cancer. J. Clin. Oncol..

[10] Aversa Z., Costelli P., Muscaritoli M. (2017). Cancer-induced muscle wasting: Latest findings in prevention and treatment. Ther. Adv. Med. Oncol..

[11] Han H.Q., Mitch W.E. (2011). Targeting the myostatin signaling pathway to treat muscle wasting diseases. Curr. Opin. Support. Palliat. Care.

[12] Zhou X., Wang J.L., Lu J., Song Y., Kwak K.S., Jiao Q., Rosenfeld R., Chen Q., Boone T., Simonet W.S. (2010). Reversal of cancer cachexia and muscle wasting by ActRIIB antagonism leads to prolonged survival. Cell.

[13] Rodriguez J., Vernus B., Chelh I., Cassar-Malek I., Gabillard J.-C., Hadj Sassi A., Seiliez I., Picard B., Bonnieu A. (2014). Myostatin and the skeletal muscle atrophy and hypertrophy signaling pathways. Cell. Mol. Life Sci..

[14] Han H., Zhou X., Mitch W.E., Goldberg A.L. (2013). Myostatin/activin pathway antagonism: Molecular basis and therapeutic potential. Int. J. Biochem. Cell Biol..

[15] MacKenzie M.G., Hamilton D.L., Pepin M., Patton A., Baar K. (2013). Inhibition of myostatin signaling through Notch activation following acute resistance exercise. PLoS ONE.

[16] Cole C.L., Kleckner I.R., Jatoi A., Schwarz E.M., Dunne R.F. (2018). The role of systemic inflammation in cancer-associated muscle wasting and rationale for exercise as a therapeutic intervention. JCSM Clin. Rep..

[17] Bowen T.S., Schuler G., Adams V. (2015). Skeletal muscle wasting in cachexia and sarcopenia: Molecular pathophysiology and impact of exercise training. J. Cachexia Sarcopenia Muscle.

[18] Kasprzak A. (2021). The role of tumor microenvironment cells in colorectal cancer (CRC) cachexia. Int. J. Mol. Sci..

[19] Directo D., Lee S.R. (2023). Cancer Cachexia: Underlying Mechanisms and Potential Therapeutic Interventions. Metabolites.

[20] Aulino P., Berardi E., Cardillo V.M., Rizzuto E., Perniconi B., Ramina C., Padula F., Spugnini E.P., Baldi A., Faiola F. (2010). Molecular, cellular and physiological characterization of the cancer cachexia-inducing C26 colon carcinoma in mouse. BMC Cancer.

[21] Moher D., Liberati A., Tetzlaff J., Altman D.G., Group P. (2010). Preferred reporting items for systematic reviews and meta-analyses: The PRISMA statement. Int. J. Surg..

[22] Macleod M.R., O’Collins T., Howells D.W., Donnan G.A. (2004). Pooling of animal experimental data reveals influence of study design and publication bias. Stroke.

[23] Tichy L., Allred K.F., Rezeli E.T., Coleman M.F., Allred C.D., Hursting S.D., Parry T.L. (2025). Concurrent Physical Activity Protects Against C26 Adenocarcinoma Tumor-Mediated Cardiac and Skeletal Muscle Dysfunction and Wasting in Males. Cells.

[24] Tsitkanou S., Koopmans P., Peterson C., Cabrera A.R., Muhyudin R., Morena F., Khadgi S., Schrems E.R., Washington T.A., Murach K.A. (2025). Myocellular adaptations to short-term weighted wheel-running exercise are largely conserved during C26-tumour induction in male and female mice. Exp. Physiol..

[25] Dalle S., Hiroux C., Koppo K. (2024). Endocannabinoid remodeling in murine cachexic muscle associates with catabolic and metabolic regulation. Biochim. Biophys. Acta (BBA)-Mol. Basis Dis..

[26] Jee H., Park E., Hur K., Kang M., Kim Y. (2022). High-intensity aerobic exercise suppresses cancer growth by regulating skeletal muscle-derived oncogenes and tumor suppressors. Front. Mol. Biosci..

[27] Fix D.K., Counts B.R., Smuder A.J., Sarzynski M.A., Koh H.J., Carson J.A. (2021). Wheel running improves fasting-induced AMPK signaling in skeletal muscle from tumor-bearing mice. Physiol. Rep..

[28] Ranjbar K., Ballarò R., Bover Q., Pin F., Beltrà M., Penna F., Costelli P. (2019). Combined exercise training positively affects muscle wasting in tumor-bearing mice. Med. Sci. Sports Exerc..

[29] Tatebayashi D., Himori K., Yamada R., Ashida Y., Miyazaki M., Yamada T. (2018). High-intensity eccentric training ameliorates muscle wasting in colon 26 tumor-bearing mice. PLoS ONE.

[30] Hardee J.P., Counts B.R., Gao S., VanderVeen B.N., Fix D.K., Koh H.J., Carson J.A. (2018). Inflammatory signalling regulates eccentric contraction-induced protein synthesis in cachectic skeletal muscle. J. Cachexia Sarcopenia Muscle.

[31] Khamoui A.V., Park B.S., Kim D.H., Yeh M.C., Oh S.L., Elam M.L., Jo E., Arjmandi B.H., Salazar G., Grant S.C. (2016). Aerobic and resistance training dependent skeletal muscle plasticity in the colon-26 murine model of cancer cachexia. Metab. Clin. Exp..

[32] Pigna E., Berardi E., Aulino P., Rizzuto E., Zampieri S., Carraro U., Kern H., Merigliano S., Gruppo M., Mericskay M. (2016). Aerobic exercise and pharmacological treatments counteract cachexia by modulating autophagy in colon cancer. Sci. Rep..

[33] Pin F., Busquets S., Toledo M., Camperi A., Lopez-Soriano F.J., Costelli P., Argilés J.M., Penna F. (2015). Combination of exercise training and erythropoietin prevents cancer-induced muscle alterations. Oncotarget.

[34] Chen M.-M., Zhao Y.-P., Zhao Y., Deng S.-L., Yu K. (2021). Regulation of myostatin on the growth and development of skeletal muscle. Front. Cell Dev. Biol..

[35] Allen D.L., Unterman T.G. (2007). Regulation of myostatin expression and myoblast differentiation by FoxO and SMAD transcription factors. Am. J. Physiol. Cell Physiol..

[36] Hildebrandt L., Dieterlen M.T., Klaeske K., Haunschild J., Saeed D., Eifert S., Borger M.A., Jawad K. (2022). Myostatin/AKT/FOXO Signaling Is Altered in Human Non-Ischemic Dilated Cardiomyopathy. Life.

[37] Oyabu M., Takigawa K., Mizutani S., Hatazawa Y., Fujita M., Ohira Y., Sugimoto T., Suzuki O., Tsuchiya K., Suganami T. (2022). FOXO1 cooperates with C/EBPδ and ATF4 to regulate skeletal muscle atrophy transcriptional program during fasting. FASEB J..

[38] Du S., Zheng H. (2021). Role of FoxO transcription factors in aging and age-related metabolic and neurodegenerative diseases. Cell Biosci..

[39] Zeng Z., Liang J., Wu L., Zhang H., Lv J., Chen N. (2020). Exercise-induced autophagy suppresses sarcopenia through Akt/mTOR and Akt/FoxO3a signal pathways and AMPK-mediated mitochondrial quality control. Front. Physiol..

[40] Yuan L., Han J., Meng Q., Xi Q., Zhuang Q., Jiang Y., Han Y., Zhang B., Fang J., Wu G. (2015). Muscle-specific E3 ubiquitin ligases are involved in muscle atrophy of cancer cachexia: An in vitro and in vivo study. Oncol. Rep..

[41] Lecker S.H., Jagoe R.T., Gilbert A., Gomes M., Baracos V., Bailey J., Price S.R., Mitch W.E., Goldberg A.L. (2004). Multiple types of skeletal muscle atrophy involve a common program of changes in gene expression. FASEB J..

