# Supplementary material for: Exercise Modulation of the Myostatin–FOXO Pathway in Murine Models of Cancer Cachexia: A Systematic Review
Source: Medicina (Kaunas). 2025 Nov 12;61(11):2022. doi: 10.3390/medicina61112022 (PMC12654131; doi:10.3390/medicina61112022)
Supplement: Supplementary file 1 [file medicina-61-02022-s001.zip › medicina-3928985-supplementary.pdf]

Supplementary Table S1. Comprehensive Search Strategy

Searches were conducted from database inception through August 2025 and were limited to English-language original experimental studies in animal models of colorectal-cancer-induced cachexia.

Database	Search String (Boolean Query)	Records Retrieved (n)
PubMed / MEDLINE	(exercise OR “physical activity” OR training) AND (myostatin OR “growth differentiation factor 8” OR GDF-8) AND (FOXO OR “forkhead box O”) AND (“muscle RING finger 1” OR MuRF-1 OR Atrogin-1 OR MAFbx) AND (“cancer cachexia” OR “tumor-induced wasting” OR “muscle atrophy” OR “colorectal cancer”)	n =7
Scopus	TITLE-ABS-KEY (exercise OR “physical activity” OR training) AND (myostatin OR “growth differentiation factor 8” OR GDF-8) AND (FOXO OR “forkhead box O”) AND (“muscle RING finger 1” OR MuRF-1 OR Atrogin-1 OR MAFbx) AND (“cancer cachexia” OR “tumor-induced wasting” OR “muscle atrophy” OR “colorectal cancer”)	n = 315
Web of Science Core Collection	TS = (exercise OR “physical activity” OR training) AND (myostatin OR “growth differentiation factor 8” OR GDF-8) AND (FOXO OR “forkhead box O”) AND (“muscle RING finger 1” OR MuRF-1 OR Atrogin-1 OR MAFbx) AND (“cancer cachexia” OR “tumor-induced wasting” OR “muscle atrophy” OR “colorectal cancer”)	n = 37
Science Direct	TITLE-ABSTR-KEY (exercise OR “physical activity” OR training) AND (myostatin OR GDF-8 OR “growth differentiation factor 8”) AND (FOXO OR “forkhead box O”) AND (MuRF-1 OR “muscle RING finger 1” OR Atrogin-1 OR MAFbx) AND (“cancer cachexia” OR “tumor-induced wasting” OR “muscle atrophy” OR “colorectal cancer”)	n = 65
